# Beyond Chronic Stress: Daily Stress Is Associated With Symptoms in Irritable Bowel Syndrome

**DOI:** 10.1016/j.cgh.2026.05.008

**Published:** 2026-05-17

**Authors:** Asal Yunusova, David J. Levinthal, Phoebe Lam, Kirk Warren Brown, Zach Branson, Sarah Wu, Bethany Sanov, Ava Liccione, Janine M. Dutcher, Emily K. Lindsay, J. David Creswell

**Affiliations:** 1 Department of Psychology, Carnegie Mellon University, Pittsburgh, Pennsylvania; 2 Division of Gastroenterology, Hepatology, and Nutrition, Department of Medicine, University of Pittsburgh Medical Center, Pittsburgh, Pennsylvania; 3 Department of Statistics and Data Science, Carnegie Mellon University, Pittsburgh, Pennsylvania; 4 Department of Psychology, University of Pittsburgh, Pittsburgh, Pennsylvania

**Keywords:** Affective Reactivity, Ecological Momentary Assessment, Gut–Brain Axis, Irritable Bowel Symptoms

## Abstract

**BACKGROUND & AIMS:**

Stress is a central factor in the pathophysiology of irritable bowel syndrome, yet little is known about how stress and symptoms fluctuate together in daily life. This study characterized real-world associations between momentary stress and symptom severity in irritable bowel syndrome, including whether stress predicts later symptoms and vice versa.

**METHODS:**

This was a prospective observational study conducted during the baseline phase of a randomized clinical trial among adults meeting Rome IV criteria for irritable bowel syndrome (N = 357). Participants completed self-report measures of chronic stress and irritable bowel syndrome symptom severity, followed by a 7-day ecological momentary assessment with 3 surveys per day assessing momentary stress and symptom severity.

**RESULTS:**

Individuals with higher average stress during the ecological momentary assessment week experienced greater irritable bowel syndrome symptom severity, and times of elevated stress relative to their usual level were linked to worse symptom severity, independent of chronic stress and symptom severity (both *P* < .001). A prospective stress–symptom association emerged during the afternoon-to-evening period; afternoon stress was associated with greater evening symptom severity (*P* = .002) and afternoon symptom severity was associated with greater evening stress (*P* = .04), whereas associations during the evening-to-next-morning transition were disrupted or inverse.

**CONCLUSIONS:**

Stress and symptoms are closely linked within the same moment, but also prospectively, particularly later in the day, among adults with irritable bowel syndrome. These findings highlight the dynamic nature of daily stress–symptom relationships and underscore the potential value of within-day stress management interventions for improving symptom control in irritable bowel syndrome.

Irritable bowel syndrome (IBS) is among the most prevalent disorders of gut–brain interaction, affecting nearly 1 in 9 people worldwide, characterized by abdominal pain related to bowel movements, bloating, urgency to defecate, and changes in bowel habits. ^1^ Recent clinical guidelines from the American Gastroenterological Association emphasize recognizing IBS as a gut–brain disorder and highlight the importance of integrating stress-management strategies.^2^ However, optimizing these approaches requires evidence on how stress influences symptoms in daily life, addressing a critical mechanistic gap in understanding the short-term dynamics of stress–symptom interactions.

Leading models highlight psychological stress—including chronic stress, life events, and early life adversity—as a potential key contributor to both the onset and persistence of IBS symptoms.^3,4^ Interestingly, symptoms can fluctuate within a single day, often worsening as the day progresses,^5,6^ yet little is known about the factors linked to these flares. It is plausible that short-term fluctuations in stress, rather than chronic stress alone, correspond with changes in IBS symptom severity. Moreover, the stress–symptom link has been previously observed as bidirectional,^7,8^ with some theoretical frameworks proposing that gastrointestinal-related anxiety in response to symptoms creates a positive feedback loop between symptoms and stress,^3^ although empirical evidence for this reciprocity in daily life remains mixed.^9–11^

The present longitudinal observational study examined how perceived psychological stress and IBS symptoms fluctuate together in daily life, linking real-time experiences to short-term gut–brain dynamics among individuals with IBS. Specifically, we hypothesized that there would be a positive relationship between: (1) baseline chronic stress and chronic IBS symptom severity; (2) both general momentary stress and overall weekly stress with momentary IBS symptom severity; and (3) a bidirectional relationship between momentary stress and future IBS symptom severity (eg, hours later). A secondary aim was to explore whether the relationship between stress and symptoms varied by time of day, that is, whether associations were stronger within a single day or between consecutive days. Together, these aims refine biopsychosocial models of IBS by elucidating short-term stress–symptom dynamics, clarifying whether the stress–symptom link is reciprocal, and informing the timing and personalization of stress-management interventions to alleviate daily symptom burden.

## Materials and Methods

### Participants

Three hundred fifty-seven adults were recruited from gastroenterology clinics, online research registry services, and flyers in the Pittsburgh area as part of a 3-month randomized controlled trial evaluating the efficacy of a mindfulness intervention for IBS (ClinicalTrials.gov, Number: NCT05083091). Results from the parent clinical trial will be reported separately; the present report focuses on preintervention data. Eligibility was initially assessed via telephone and later transitioned to an online survey to improve recruitment efficiency; however, study team members contacted participants by phone or email when additional eligibility clarification was needed. Although participants reported whether they had an existing IBS diagnosis, eligibility was determined based on meeting the Rome IV IBS criteria^12^ at the time of screening. Participants were also required to exhibit at least mild psychological distress (a score of at least a 3 on the Patient Health Questionnaire^13^). Participants with inflammatory bowel disease and bowel cancer were automatically excluded, whereas other reported gastrointestinal medical history was reviewed by study team members and a physician investigator to determine final eligibility on a case-by-case basis. Health conditions were additionally collected via a free-response item at baseline for descriptive purposes and were not used for eligibility determination. Enrollment was open to individuals of all sexes and gender identities.

### Study Design

Participants attended an in-person baseline session that included informed consent, a confirmatory eligibility check (to ensure no major treatment changes since screening), baseline questionnaires, and installation and instructions for the ecological momentary assessment (EMA) phone application (‘Wear-it’^14^). The Rome IV questionnaire was administered at baseline for descriptive purposes and was not used for eligibility determination, as it was expected that responses would remain consistent between the time of screening and baseline session. Following the session, participants completed EMAs measuring stress and IBS symptom severity over a 7-day preintervention observational period. Carnegie Mellon’s Institutional Review Board approved the parent trial in March 2021, and data collection was completed in February 2025 (see ClinicalTrials.gov for details).

### Measures

#### EMA outcomes.

At each EMA prompt, participants rated their current perceived stress with the item, “Right now, how much general stress are you experiencing or feeling?” rated on a 1 (no stress) to 7 (severe stress) scale,^15^ and their current IBS symptom severity with a single global item, “Right now, how severe are your IBS symptoms?” rated 1 (not at all severe) to 7 (extremely severe). A single global symptom item was used to balance measurement precision with participant burden, given the high assessment frequency. Higher scores indicate greater momentary stress and IBS symptom severity, respectively. Analyses used both momentary ratings and each participants’ average across the 21 possible EMAs.

#### Time-related variables.

Time of day (morning, afternoon, evening), day of study (0–6), and weekday vs weekend were derived from EMA timestamps, survey identifiers (Daily 1, 2, 3), and participant study timelines. EMAs were delivered quasirandomly within 4-hour time windows, beginning 1 hour after each participant’s usual wake time, with at least 105 minutes between surveys, and were available for 1 hour.

#### Demographic and clinical characteristics.

Participants reported their age, race, sex assigned at birth, gender identity (female, male, nonbinary or genderqueer, or prefer not to say), education level, and IBS subtype (constipation-predominant [IBS-C], diarrhea-predominant [IBS-D], mixed bowel habits [IBS-M]), years living with IBS symptoms and years since diagnosis, and other health conditions. Education was recoded into 4 categories (less than college, some college, college graduate, advanced degree). One individual who did not disclose their gender was grouped with individuals identifying as nonbinary or genderqueer.

#### Chronic stress and symptom severity.

Chronic stress and symptoms were measured with the 10-item Perceived Stress Scale^16^ and the 5-item IBS Symptom Severity Scale.^17^ Higher scores indicate greater chronic stress over the past month and IBS severity over the past 10 days.

### Statistical Analysis

The original analytic sample (N = 360) was based on a priori power analysis for the parent trial. Pearson correlations tested the association between chronic stress and symptom severity. Multilevel models evaluated same-moment associations between stress and symptom severity across repeated smartphone assessments. Each model simultaneously included 2 components of stress derived from the EMA stress item: momentary stress relative to a person’s own average and each person’s average stress across the week. Parsing stress into these 2 components was critical because it distinguished within-person fluctuations from between-person differences. Covariates included time (time of day, study day, weekday vs weekend), participant characteristics (age, gender, sex, education, and IBS subtype), and chronic stress and symptom severity.

After testing these cross-sectional associations, multilevel models were used to evaluate longitudinal links, examining whether stress at 1 EMA was associated with symptom severity at the next scheduled EMA (morning to afternoon, afternoon to evening, and evening to the next morning). In these models, momentary stress was lagged such that morning stress predicted afternoon symptom severity, and so on. Because longitudinal associations can only occur at the within-person level, lagged momentary stress was the primary predictor, whereas average stress was included as a covariate to account for stable between-person differences, along with all other covariates listed above. Reverse models were also estimated to evaluate whether symptom severity predicted subsequent stress. The main text reports fully adjusted models, whereas sequential model-building steps are presented in the Supplementary Materials to illustrate how covariate adjustment influences estimates. Finally, exploratory time-of-day moderation analyses tested whether the strength of these associations differed across assessment periods.

Analyses were conducted in R Studio^18,19^ (nlme package^20^) with restricted maximum likelihood, random intercepts for participants, and random slopes for momentary predictors. Significant interaction effects were followed by simple slope analyses, and the overall significance of interaction terms was evaluated using Wald *F*-tests. Primary analyses were hypothesis-driven and therefore not corrected for multiple comparisons; exploratory analyses are explicitly labeled and interpreted cautiously (see https://osf.io/6qft9 and Supplementary Table 1 for preregistration details). Results are reported as unstandardized B (SE) with 95% confidence interval (CI), with statistical significance defined as *P* < .05.

## Results

Three hundred fifty-seven participants (mean age, 34.22 years; standard deviation [SD], 13.74 years) completed the in-person baseline assessment. Most identified as female gender (79.0%), female sex assigned at birth (84.3%), and Caucasian/White (81.2%). Educational level ranged from General Education Development to doctoral/professional degrees. Self-reported IBS subtypes included IBS-M (54.3%), IBS-D (25.8%), and IBS-C (19.9%). Three hundred twenty-three participants (90.5%) still met the Rome IV criteria at baseline. On average, participants had an IBS diagnosis for 5.75 years (SD, 3.59 years; n = 12 missing) and symptoms for 7.92 years (SD, 2.91 years). Compliance was high; 76.5% completed ≥80% of EMA stress ratings (n = 273), and 77% completed ≥80% of symptom ratings (n = 275). Within-person consistency across days for momentary stress and symptom severity was high (α = .88; α = .86), and the Perceived Stress Scale showed good internal reliability (α = .81). Intraclass correlations indicated substantial within-person variance in stress (61.4%) and symptom severity (65.1%), motivating the decomposition of predictors into within- and between-person components. The Supplementary Materials provide a participant flowchart (Supplementary Figure 1), full baseline descriptives (Supplementary Tables 2 and 3), zero-order correlations (Supplementary Table 4), missing data details (Supplementary Table 5), and individual participant trajectories (Supplementary Figure 2 and 3).

### Are chronic stress and symptom severity correlated?

Consistent with prior research, ^21^ greater chronic stress on the Perceived Stress Scale was significantly associated with greater chronic symptom severity on the IBS Symptom Severity Scale (*r* = 0.11; *P* = .03).

### Is average stress and momentary stress related to momentary IBS symptom severity?

**Figure 1.**
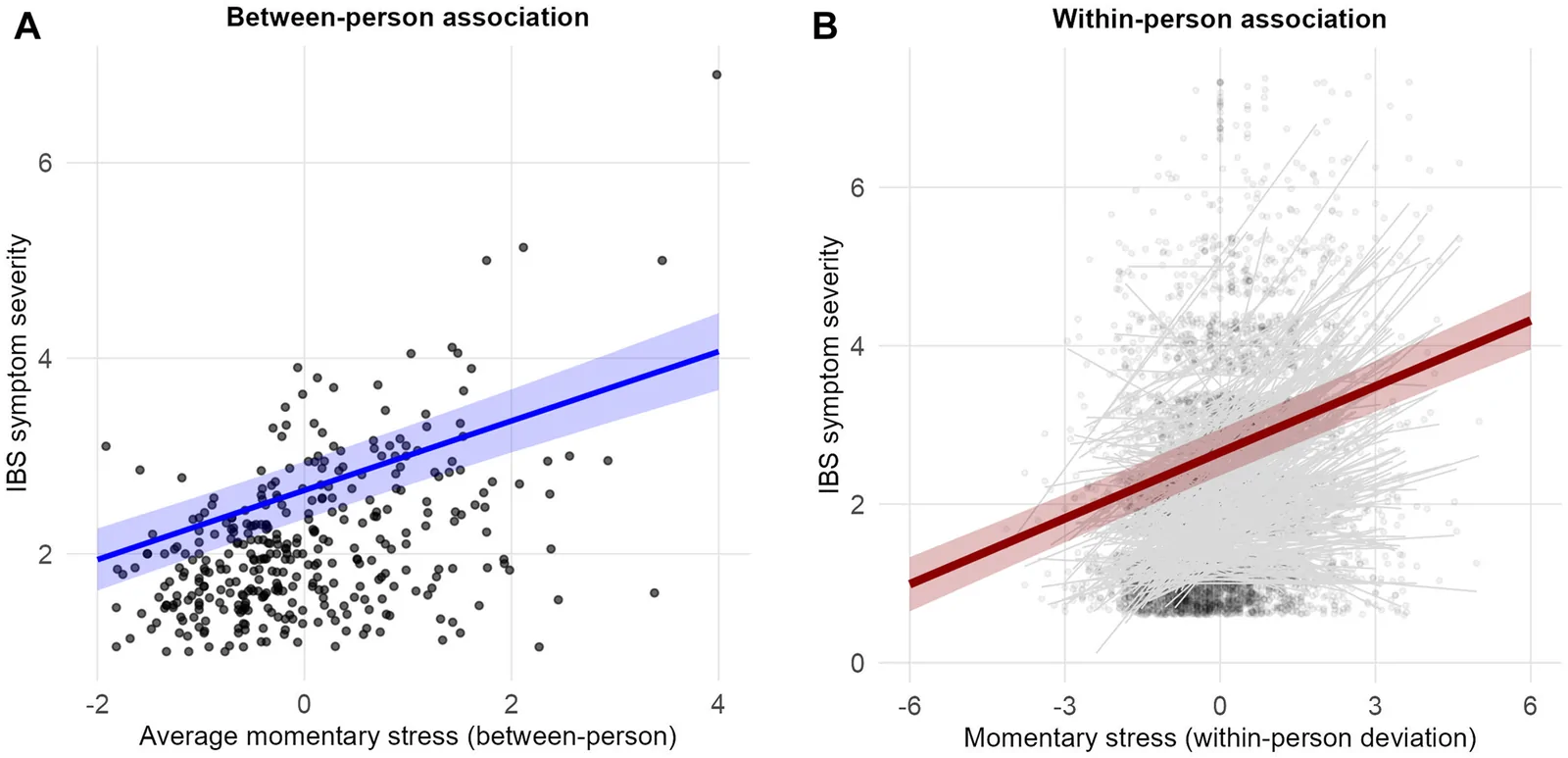
Between-person and within-person association between stress and symptom severity. Panel *A* shows the between-person association, where the x-axis represents each participant’s mean stress across days (grand-mean–centered). The *blue line* shows the model-estimated between-person association (95% CI), reflecting whether individuals with higher average stress report higher overall symptom severity. Panel *B* shows the within-person association, where stress was person-mean–centered such that the x-axis represents momentary deviations from an individual’s own average stress. The *red line* shows the model-estimated within-person association (95% CI), with *light gray lines* indicating individual participant slopes. Estimates are derived from the concurrent multilevel model reported in Table 1.

Across participants, higher average stress during the week was associated with greater momentary symptom severity (b, 0.35; 95% CI, 0.29–0.42; *P* < .001) (Figure 1A). In the same model, within individuals, moments of higher-than-usual stress were also associated with greater symptom severity at that same moment (b, 0.28; 95% CI, 0.24–0.31; *P* < .001, (Figure 1B; Table 1).

### Is prior stress related to subsequent symptom severity and vice versa?

**Figure 2.**
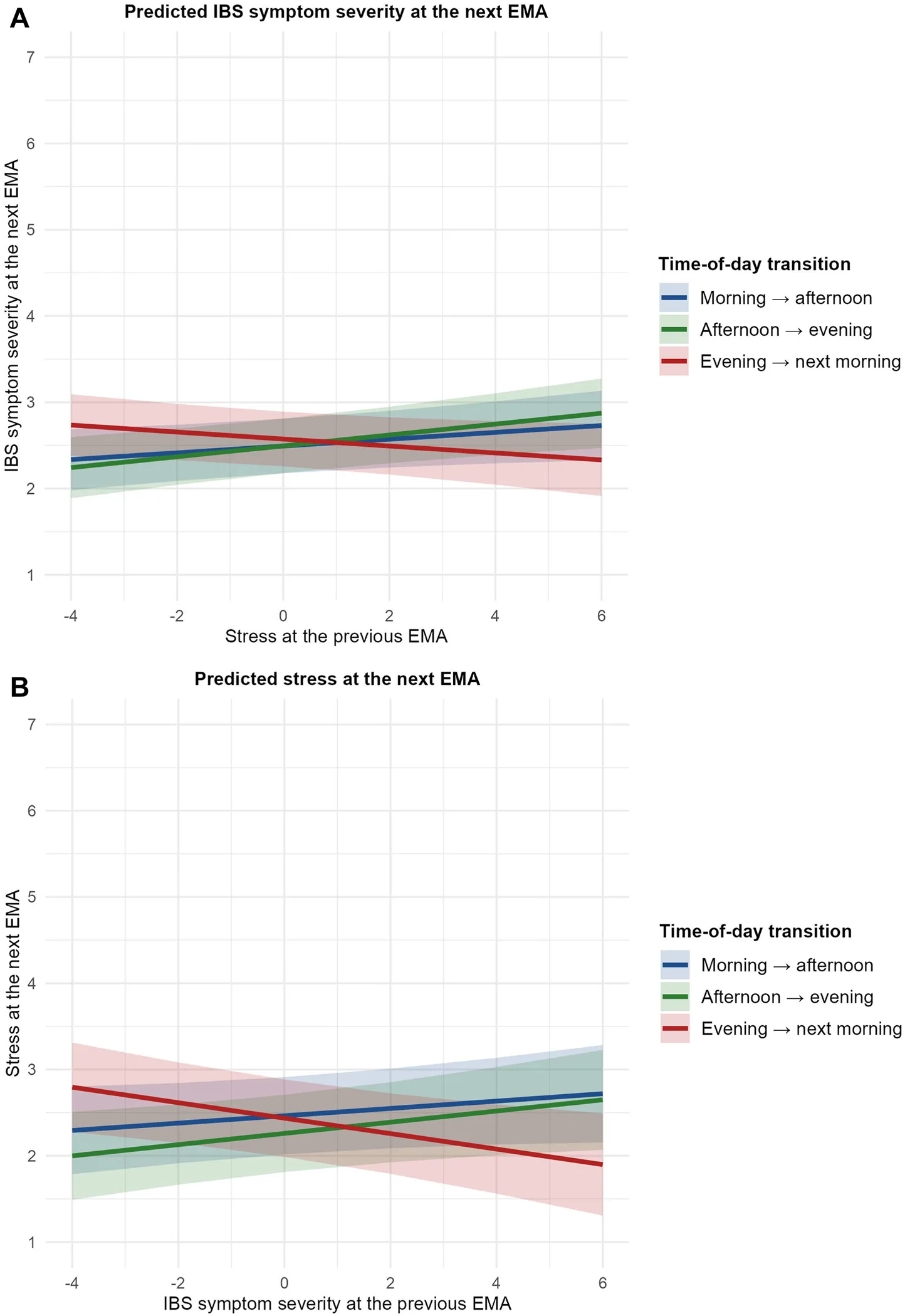
Bidirectional prospective associations between stress and symptom severity across time-of-day transitions. Panel *A* shows the prospective within-person association between prior stress and subsequent symptom severity, where prior stress was person-mean–centered such that the x-axis represents deviations from an individual’s own average stress at the previous assessment. Panel *B* shows the reverse association, where prior person-mean–centered symptom severity predicts subsequent stress. In both panels, person-level mean stress (or symptom severity) was included to account for stable between-person differences. Panel *A* corresponds to the time-of-day moderation model reported in Table 1, and Panel *B* corresponds to the time-of-day moderation model reported in Table 2.

The adjusted lagged association between stress and subsequent symptom severity was positive but imprecise (b, 0.02; 95% CI, − 0.00 to 0.05; *P* = .07) (Table 2), as the CI included zero. However, this association varied significantly by time of day (χ^2^(2) = 13.43; *P* = .001) (see Figure 2A; Table 1 for pairwise contrasts). Simple slope tests indicated that afternoon stress was associated with greater evening symptom severity (b, 0.06; SE, 0.02; 95% CI, .02−0.10; *P* = .002). In contrast, evening-to-next-morning (b, − 0.04; SE, 0.02; 95% CI, −0.08 to 0.00; *P* = .07), and morning-to-afternoon slopes (b, 0.04; SE, 0.02; 95% CI, −0.00 to 0.08; *P* = .06) had CIs that included zero.

In the reverse direction, there was no association between symptom severity and subsequent stress (b, 0.01; 95% CI, −0.03 to 0.05; *P* = .57). Once again, time of day influenced the association (χ^2^(2) = 14.42; *P* < .001) (see Figure 2B; Table 2 for pairwise contrasts). Simple slope tests revealed a similar pattern as above, afternoon symptom severity was associated with higher evening stress (b, 0.07; SE, 0.03; 95% CI, 0.00–0.13; *P* = .04), whereas the morning-to-afternoon slope did not provide evidence for a lagged association (b, 0.04; SE, 0.03; 95% CI, −0.02 to 0.10; *P* = .15). Evening symptom severity was associated with lower stress the following morning (b, −0.09; SE, 0.03; 95% CI, −0.15 to −0.03; *P* = .01).

## Discussion

Pathophysiological accounts of IBS have long proposed that psychosocial stress, including chronic life stress and threatening life events, contributes to both the onset and maintenance of IBS symptoms,^3,22,23^ a pattern supported by the present findings, which replicated the well-established cross-sectional association between chronic stress and symptom severity.^21^ However, it has remained unclear whether stress and symptom severity are bidirectionally associated in daily life, and more specifically, whether these associations reflect within-person dynamics or stable differences between individuals.

Although prior work^11^ has demonstrated positive person-specific associations between stress and symptoms using idiographic analyses, these models did not account for between-person differences or chronic stress and symptom severity. Above and beyond time-related factors, sociodemographic characteristics, IBS subtype, and chronic stress and symptom severity, current findings suggest that individuals who, on average, experienced a more stressful week also tended to report higher momentary symptom severity in daily life. Importantly, beyond these between-person differences, stress and symptoms were also dynamically coupled within individuals: individuals reported more severe IBS symptoms during moments of higher stress relative to their own average.

Beyond co-occurring increases in stress and symptoms, a key question is whether stress precedes symptoms. Experimental studies manipulating stress have shown that psychological stress can exert immediate or delayed effects on gastrointestinal symptoms.^24^ Building on this laboratory evidence, prior EMA and daily diary studies^9–11,25–31^ have leveraged intensive longitudinal designs to test temporal ordering and prospective associations, examining whether stress prospectively predicts subsequent symptoms in daily life and vice versa. In the current study, although preliminary lagged models showed significant associations in both directions (Supplementary Tables 7–8), after accounting for all covariates, the association between stress and subsequent symptom severity remained positive but did not reach statistical significance, whereas symptom severity was not associated with subsequent stress. However, converging exploratory analyses indicated that the stress–symptom coupling may be temporally constrained, emerging more strongly later in the day (afternoon to evening), with little evidence that these positive associations persist across the overnight period. These temporal dynamics may partly reflect features of the measurement design, such that longer lags, or more hours elapsed between surveys, weaken associations between stress and symptoms. Alternatively, they may reflect underlying circadian processes and the cumulative buildup of stress and symptoms across the day.

Circadian rhythm processes have been increasingly implicated in IBS, with lifestyle habits such as sleep and feeding times influencing digestive physiology.^32–35^ Symptom expression also varies across the day, with reports of bloating worsening later in the day,^5,6^ as 1 example (compare^36^). The salience of the afternoon-to-evening period may reflect the accumulation of daytime stressors, which may heighten vulnerability to symptom flares as the day progresses, although a smaller review of 5 EMA studies found inconsistent associations between time of day and stress across different populations.^37^ Although mechanistic interpretations are limited without symptom-specific measures (eg, diarrhea vs pain-related severity), these preliminary findings suggest that stress–symptom coupling may not be uniform across the day, with the afternoon-to-evening period emerging as a possible key window for intervention and for understanding processes that may accumulate earlier in the day to trigger later symptom flares. However, the presence of a small potential lagged effect during the morning-to-afternoon period, as suggested by the CIs, warrants further investigation before concluding that the afternoon-to-evening window is uniquely sensitive.

In contrast to within-day patterns, stress–symptom dynamics between consecutive days showed an inverse association. Greater evening symptoms were significantly associated with lower next-morning stress. The negative beta and corresponding CI for the evening-to-morning stress → symptom slope further suggested a small, lagged effect. Prior research on cross-day stress–symptom associations has yielded mixed results.^9,26,28,29,31^ Together, these findings shed light on possible nightly recovery processes that may temporarily reset stress–symptom dynamics. There is growing evidence that poor sleep may increase the risk of IBS and worsen symptoms,^38–41^ and some evidence of reciprocal patterning, such that days of heightened IBS symptoms are associated with improved subsequent sleep efficiency.^42^ Moreover, patients with sleep disorders and pre-existing psychological distress have shown a higher risk of developing IBS and a greater sensitivity for developing psychological distress compared with other comorbid conditions.^8^ Without the availability of objective sleep measures, the current study could not further test the role of sleep. Thus, the possible role of restorative sleep in shaping stress–symptom relationships remains speculative, representing an important future direction for research.

Although causality cannot be determined from this study, there was evidence that individuals who experienced particularly stressful moments also tended to report higher symptom severity at that same moment, and there was preliminary evidence that prospective associations between stress and symptoms may vary by time of day. The within-day bidirectional associations observed in the present data are consistent with a broader literature suggesting reciprocal links between stress and IBS, including experimental stress-induction studies,^24^ longitudinal evidence identifying psychosocial risk markers for IBS onset,^43,44^ and epidemiologic findings indicating that IBS increases risk for newly diagnosed psychiatric disorders.^45^ Importantly, these results should not be taken to imply that symptoms are always driven by stress; symptom flares may arise from other triggers (eg, diet^46^) or behavioral factors (eg, disturbed sleep, sedentary behavior^39^). This, in turn, may elevate stress and perpetuate a positive feedback loop of symptom-related anxiety and physiological arousal, as suggested by other models.^4^ Overall, it is important to interpret these findings cautiously in the context of the beta coefficients, which were small in magnitude. Further research is needed to more precisely estimate the magnitude and clinical significance of these associations and to determine whether time-of-day variation is replicated in independent samples.

To our knowledge, this is the first study to disentangle between-person and within-person associations to characterize how stress and IBS symptoms may be linked in daily life. By sampling stress and symptom severity multiple times per day over 1 week, the study provides a real-world view of these processes, enhancing ecological validity and enabling the examination of short-term temporal dynamics that are difficult to capture using retrospective or cross-sectional methods. Although time-of-day moderation analyses were exploratory, they served a hypothesis-generating role by revealing temporally specific stress–symptom patterns, illustrating how moderation can uncover heterogeneity in daily dynamics and guide future research on circadian and sleep-related processes. In addition to these strengths, several limitations should also be noted. The assessment period was limited to 1 week. Rome IV criteria were not rechecked at baseline, which may have introduced some diagnostic misclassification; however, prior work suggests only moderate test–retest reliability of the Rome IV diagnostic questionnaire.^12^ Additionally, IBS subtype was self-reported and not confirmed with Rome IV criteria. The relatively high prevalence of mixed IBS may further constrain generalizability. There may also be selection bias, as participants were enrolling in a behavioral intervention trial and may have heightened awareness of the stress–symptom link. However, patients with IBS are often quite aware of the potential role of stress, frequently citing it as having a greater influence on their symptoms than other factors.^47^ Furthermore, the study relied on a single global EMA item to assess IBS symptom severity and did not assess specific momentary stressors or recent life events, limiting the ability to contextualize stress reports, draw symptom-specific inferences, or identify potential mechanisms. Future research should consider incorporating such measurements. Finally, the observational design precludes causal inference.

## Conclusions

These results suggest that momentary stress may represent a relevant process associated with daily symptom fluctuations in IBS, particularly within the same day. Compared with other putative contributors to IBS symptoms (eg, gut microbiome,^48,49^ visceral sensitivity^50^), stress may represent a modifiable target with short-term dynamics that can be monitored in real time. Digital health tools that track stress and symptoms throughout the day may help patients and providers explore individualized patterns and potential triggers, whereas just-in-time adaptive interventions^51^ could be evaluated in future work as a means of delivering stress reduction strategies at moments of elevated stress. Beyond stress reduction, interventions targeting maladaptive cognitions about gastrointestinal sensations, such as cognitive behavioral therapy^52^ or mindfulness-based approaches,^53,54^ may help attenuate reinforcing cycles of stress and symptom severity. Embedding these strategies into daily routines offers a promising direction for personalized symptom management in IBS, although further confirmatory studies are needed to clarify stress–symptom dynamics in daily life.

## Supplementary Material

Supplementary Materials

Note: To access the supplementary material accompanying this article, visit the online version of *Clinical Gastroenterology and Hepatology* at www.cghjournal.org, and at https://doi.org/10.1016/j.cgh.2026.05.008.

## Figures and Tables

**Table 1:** Estimates of Concurrent and Prior Stress on Symptom Severity

Predictors	Concurrent stress-symptoms	Stress predicting symptoms	Stress predicting symptoms (time of day)
Afternoon (vs morning)	**−0.07 (0.02); *P* = .003** ^a^	**−0.09 (0.03); *P* = .01** ^a^	**−0.08 (0.03); *P* = .01** ^a^
Evening (vs morning)	−0.03 (0.03); *P* = .29	**−0.09 (0.03); *P* = .01** ^a^	**−0.08 (0.03); *P* = .02** ^a^
Day	**0.02 (0.01); *P* = .001** ^a^	**0.03 (0.01); *P* < .001** ^a^	**0.03 (0.01); *P* < .001** ^a^
Weekday (vs weekend)	**−0.08 (0.03); *P* = .004** ^a^	0.00 (0.03); *P* = .96	0.00 (0.03); *P* = .93
Age, *y*	**−0.01 (0.00); *P* = .01** ^a^	**−0.01 (0.00); *P* = .02** ^a^	**−0.01 (0.00); *P* = .02** ^a^
Female gender (vs male)	−0.01 (0.20); *P* = .98	−0.02 (0.22); *P* = .94	−0.02 (0.22); *P* = .93
Nonbinary, genderqueer, or nondisclosed gender (vs male)	−0.19 (0.22); *P* = .40	−0.26 (0.24); *P* = .28	−0.27 (0.24); *P* = .26
Female sex at birth (vs male)	−0.11 (0.20); *P* = .60	−0.16 (0.21); *P* = .46	−0.16 (0.21); *P* = .47
Some college (vs high school)	−0.05 (0.13); *P* = .72	0.02 (0.14); *P* = .87	0.02 (0.14); *P* = .88
College graduate (vs high school)	−0.13 (0.11); *P* = .27	0.00 (0.12); *P* = .98	−0.01 (0.12); *P* = .96
Advanced degree (vs high school)	−0.20 (0.12); *P* = .11	−0.11 (0.13); *P* = .40	−0.11 (0.13); *P* = .39
IBS–D (vs IBS–C)	**−0.42 (0.09); *P* < .001** ^a^	**−0.46 (0.10); *P* < .001** ^a^	**−0.46 (0.10); *P* < .001** ^a^
IBS–M (vs IBS–C)	**−0.24 (0.08); *P* = .004** ^a^	**−0.23 (0.09); *P* = .01** ^a^	**−0.23 (0.09); *P* = .01** ^a^
Chronic stress	**−0.01 (0.01); *P* = .02** ^a^	**−0.02 (0.01); *P* = .003** ^a^	**−0.02 (0.01); *P* = .004** ^a^
Chronic symptom severity	**0.00 (0.00); *P* < .001** ^a^	**0.00 (0.00); *P* < .001** ^a^	**0.00 (0.00); *P* < .001** ^a^
Prior severity	–	**0.13 (0.02); *P* < .001** ^a^	**0.13 (0.02); *P* < .001** ^a^
Average stress	**0.35 (0.03); *P* < .001** ^a^	**0.34 (0.04); *P* < .001** ^a^	**0.34 (0.04); *P* < .001** ^a^
Momentary stress	**0.28 (0.02); *P* < .001** ^a^	–	–
Prior stress	–	0.02 (0.01); *P* = .07^b^	−0.04 (0.02); *P* = .07^b^
Prior stress x morning to afternoon (vs evening to next morning)	–	−	**0.08 (0.03); *P* = .01** ^a^
Prior stress x afternoon to evening (vs evening to next morning)	–	–	**0.10 (0.03); *P* < .001** ^a^

NOTE. The concurrent and lagged models included 6307 observations (n = 352) and 5340 observations (n = 350), respectively.

See Supplementary Tables 6 and 7 for sequential models.

NOTE. Boldface values indicate statistical significance.

IBS, irritable bowel syndrome; IBS-C, constipation-predominant IBS; IBS-D, diarrhea-predominant IBS; IBS-M, mixed bowel habits IBS.

a *P* < .05.

b *P* < .10.

**Table 2:** Estimates of Prior Symptom Severity on Stress

Predictors	Symptoms predicting stress	Symptoms predicting stress (time of day)
Afternoon (vs morning)	0.03 (0.04); *P* = .51	0.03 (0.04); *P* = .49
Evening (vs morning)	**–0.18 (0.04); *P* < .001** ^a^	**–0.18 (0.04); *P* < .001** ^a^
Day	**0.03 (0.01); *P* = .002** ^a^	**0.03 (0.01); *P* = .002** ^a^
Weekday (vs weekend)	**0.24 (0.04); *P* < .001** ^a^	**0.24 (0.04); *P* < .001** ^a^
Age, *y*	0.00 (0.00); *P* = .95	0.00 (0.00); *P* = .95
Female gender (vs male)	0.25 (0.31); *P* = .42	0.25 (0.31); *P* = .42
Nonbinary, genderqueer, or nondisclosed gender (vs male)	0.50 (0.34); *P* = .15	0.49 (0.34); *P* = .15
Female sex at birth (vs male)	−0.19 (0.30); *P* = .52	−0.19 (0.30); *P* = .53
Some college (vs high school)	0.07 (0.19); *P* = .72	0.07 (0.20); *P* = .72
College graduate (vs high school)	0.24 (0.17); *P* = .17	0.24 (0.17); *P* = .17
Advanced degree (vs high school)	0.35 (0.18); *P* = .06^b^	0.35 (0.18); *P* = .06^b^
IBS-D vs IBS-C	**0.35 (0.15); *P* = .02** ^a^	**0.35 (0.15); *P* = .02** ^a^
IBS-M vs IBS-C	0.12 (0.13); *P* = .35	0.12 (0.13); *P* = .35
Chronic stress	**0.05 (0.01); *P* < .001** ^a^	**0.05 (0.01); *P* < .001** ^a^
Chronic symptom severity	0.00 (0.00); *P* = .36	0.00 (0.00); *P* = .36
Prior stress	**0.17 (0.02); *P* < .001** ^a^	**0.17 (0.02); *P* < .001** ^a^
Average symptom severity	**0.65 (0.07); *P* < .001** ^a^	**0.65 (0.07); *P* < .001** ^a^
Prior severity	0.01 (0.02); *P* = .57	**–0.09 (0.03); *P* = .01** ^a^
Prior symptom severity x morning to afternoon (vs evening to next morning)	–	**0.13 (0.04); *P* = .002** ^a^
Prior symptom severity x afternoon to evening (vs evening to next morning)	–	**0.15 (0.04); *P* < .001** ^a^

NOTE. Both models included 5327 observations (n = 350). See Supplementary Table 8 for sequential models.

NOTE. Boldface values indicate statistical significance.

IBS, irritable bowel syndrome; IBS-C, constipation-predominant IBS; IBS-D, diarrhea-predominant IBS; IBS-M, mixed bowel habits IBS.

a *P* < .05.

b *P* < .10.

## Data Availability

Data from the parent trial are currently under analysis and therefore not publicly available. Data, analytic methods, and study materials supporting the findings of the present study may be made available upon reasonable request to the corresponding author.

## What You Need to Know

### Background

Chronic stress is recognized as a key trigger of irritable bowel syndrome symptoms, but prior research has not clarified how stress relates to symptom changes as people go about their daily lives.

### Findings

Real-time tracking showed that both higher overall stress and short-term stress spikes were linked to more severe irritable bowel syndrome symptoms. Stress increases predicted same-day symptom flareups, particularly from afternoon to evening.

### Implications for patient care

Daily stress monitoring may help identify periods of heightened symptom vulnerability. Timely stress-management strategies could improve daily symptom control and overall quality of life in patients with irritable bowel syndrome.

## Abbreviations used in this paper:

CI: confidence interval
EMA: ecological momentary assessment
IBS: irritable bowel syndrome
IBS-C: constipation-predominant IBS
IBS-D: diarrhea-predominant IBS
IBS-M: mixed bowel habits IBS
SD: standard deviation
