# Supplementary material for: Beyond Chronic Stress: Daily Stress Is Associated With Symptoms in Irritable Bowel Syndrome
Source: Clin Gastroenterol Hepatol. Author manuscript; Available in PMC 2026 Sep 24. (PMC13604563; doi:10.1016/j.cgh.2026.05.008)
Supplement: Supplementary file 1 [file NIHMS2207217-supplement-Supplementary_Materials.pdf]

Supplementary Materials

In line with recent recommendations for transparent reporting, we provide a summary table describing final data preprocessing and analyses that were updated from the most up to date OSF preregistration for the hypotheses examined in this manuscript. This structured format, first proposed by Willroth and colleagues[1] and now increasingly adopted in empirical reports, improves clarity and reproducibility by distinguishing planned analyses from analytic refinements. Results from additional preregistered analyses not directly related to the present hypotheses will be reported separately.

Table S1.
Open science and transparency report focused on describing practices that deviated from the pre-registration.

<table>
<tr><th>#</th><th colspan="2">Details</th><th>Original Wording</th><th>Deviation Description</th><th>Reader Impact</th></tr>
<tr><td rowspan="3">1</td><td>Type</td><td>Hypotheses</td><td rowspan="3">Hypothesis: Greater momentary general stress and IBS distress at time t will predict greater momentary IBS symptom severity at time t+1 and vice versa. There will also be a positive concurrent relation between these variables at the within and between subject level.</td><td rowspan="3">IBS distress (“Right now, my IBS symptoms are causing me distress”) was excluded from primary analyses.</td><td rowspan="3">Models including EMA distress were not reported due to multicollinearity between IBS distress and symptom severity (r = .83).

Note that the concurrent hypothesis was added in an update. Because the pre-registered prospective link was still reported, adding the concurrent link allows readers to evaluate whether nonsignificant prospective change links reflected a lack of association per se or a lack of association in symptom change.</td></tr>
<tr><td>Reason</td><td>New knowledge and Typo/Error</td></tr>
<tr><td>Timing</td><td>After results known</td></tr>
<tr><td rowspan="3">2</td><td>Type</td><td>Data Preparation</td><td rowspan="3">Non-normal continuous variables would be transformed.</td><td rowspan="3">Continuous predictors were not transformed.</td><td rowspan="3">Multilevel modeling does not require normality and this choice retains interpretability of scales in original units.</td></tr>
<tr><td>Reason</td><td>New knowledge</td></tr>
<tr><td>Timing</td><td>After results known</td></tr>
</table>

[1] Willroth EC, Atherton OE. Best Laid Plans: A Guide to Reporting Preregistration Deviations. Advances in Methods and Practices in Psychological Science. 2024 Jan 1;7(1):25152459231213802.

3	Type	Analysis	The original preregistration implied that each hypothesis would be tested using a single, fully adjusted model. Covariates included gender, sex, IBS subtype, age, time (day, day of week, time of day), the lagged dependent variable (e.g., symptom severity at time *t–1* when predicting severity at time *t*), and concurrent predictor (e.g., stress at time *t* when predicting severity at time *t*).	Additional models were run with increasing covariate adjustment. Between-person average stress and average symptom severity, which were not explicitly specified in the preregistration, were included as between-subject covariates. Concurrent predictors were not included in lagged models; instead, models controlled for the outcome at time *t*, consistent with a prospective change framework.	All final models included the full set of preregistered covariates; intermediate models are presented to illustrate the robustness of effects across model specifications. Inclusion of between-person average stress and symptom severity provides a more stringent test of preregistered lagged associations.
	Reason	Typo/Error			
	Timing	After results known			
4	Type	Analysis	The original preregistration used G*power analysis for predictions.	We decided to run analyses on the full sample size, after data collection was complete, rather than stopping at the sample size that was suggested by the a priori power calculations.	G*Power is not designed for multilevel models, therefore our preregistered power approach was not fully applicable to the multilevel analyses. As a result, our a priori power evidence for those models is weaker than planned, and the detectable-effect thresholds are less precise. Collectively, these deviations affect certainty about minimum detectable effects, but do not alter the prespecified hypotheses, analytic specification, or the transparency of reported results.
	Reason	Typo/Error			
	Timing	After results known			

Note. The original preregistration was submitted on May 4, 2023, then updates were made on November 27, 2023, March 6, 2024, and January 23, 2025; all versions and time-stamped updates are publicly available on OSF. Deviations summarized in this table are evaluated relative to the final preregistration version that preceded analysis of the EMA data.

Figure S1. Participant flow diagram.

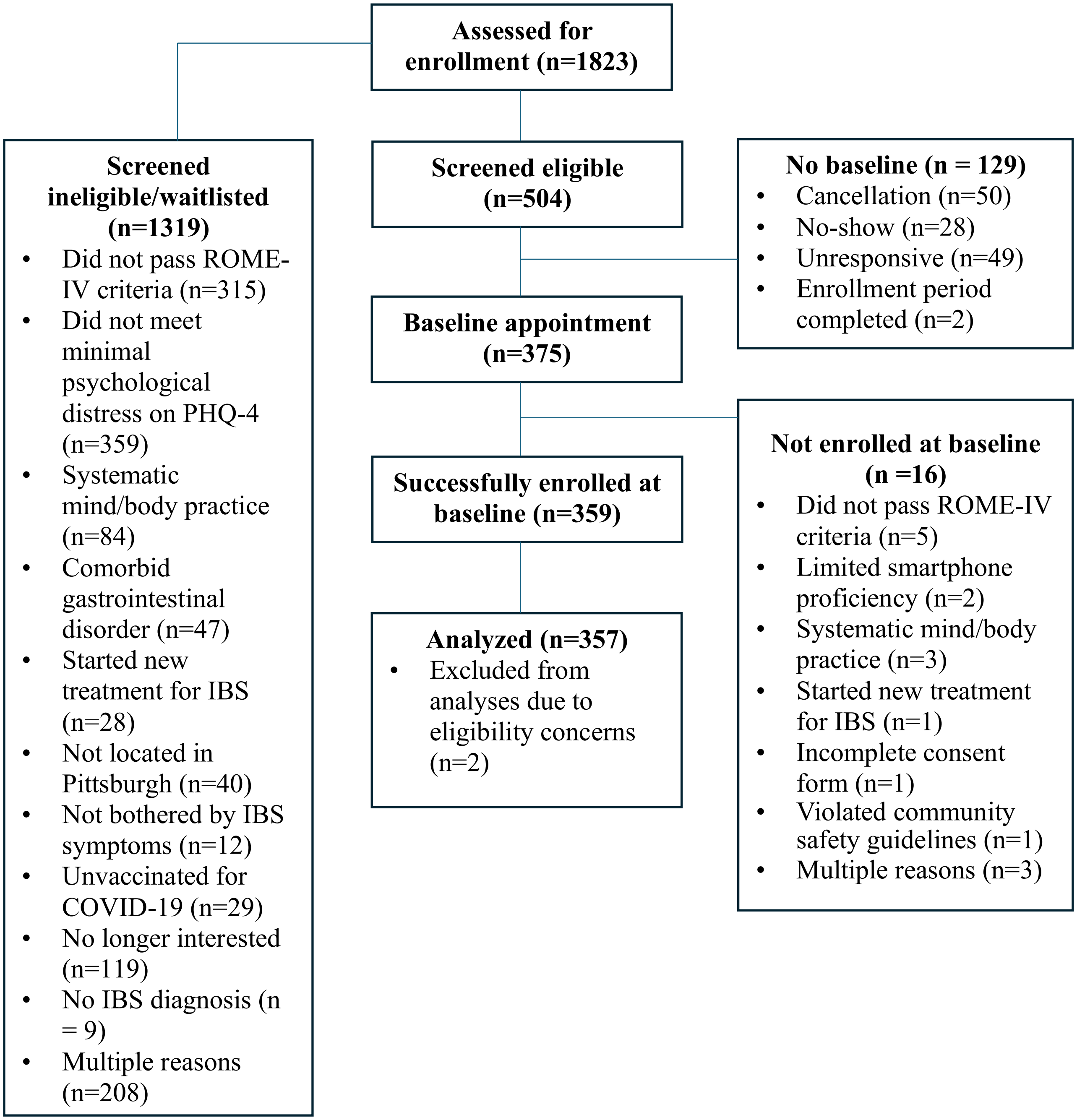

Note. Systematic mind–body practice was defined as regular mindfulness-related activity exceeding 60 minutes per week. During screening, participants described their typical stress-reduction techniques to assess engagement and level of knowledge with mindfulness practices. Eligibility initially required participants to have an IBS diagnosis, but this requirement was later removed provided participants met ROME IV IBS diagnostic criteria.

Table S2. Participant characteristics at baseline.

Variable	n (%) or Mean (SD)
Gender	
Female	282 (79.0%)
Male	53 (14.8%)
Nonbinary or genderqueer	21 (5.9%)
Prefer not to say	1 (0.3%)
Sex	
Female	301 (84.3%)
Male	56 (15.7%)
Race	
Caucasian/White	290 (81.2%)
African American/Black	23 (6.4%)
Biracial or multiracial	15 (4.2%)
South Asian	11 (3.1%)
East Asian	8 (2.2%)
Other	8 (2.2%)
Native American/American Indian	1 (0.3%)
Unreported	1 (0.3%)
Education	
General Education Development (GED)	3 (0.8%)
High school diploma	26 (7.3%)
Technical training	4 (1.1%)
Some college, no degree	62 (17.4%)
Associate degree	26 (7.3%)
Bachelor's degree	136 (38.1%)
Master's degree	75 (21.0%)
MD/PhD/JD/PharmD	25 (7.0%)
IBS subtype	
IBS-M	194 (54.3%)
IBS-D	92 (25.8%)
IBS-C	71 (19.9%)
Health conditions	
Gastrointestinal conditions and surgeries	6 (1.7%)
Neurologic and chronic pain conditions	29 (8.1%)
Inflammatory conditions	27 (7.6%)
Psychological conditions	15 (4.2%)
Other health conditions	9 (2.5%)
One or more of the above	110 (30.8%)
None	161 (45.1%)
Trait mindfulness total	74.58 (11.29)
Distress tolerance total	3.00 (0.86)
Childhood trauma history total	54.81 (7.85)

Note. Trait mindfulness, distress tolerance, and childhood trauma were assessed using the Five Facet Mindfulness Questionnaire (FFMQ; 24 items; Baer et al., 2006)[2], the Distress Tolerance Scale (DTS; 15 items; Simons & Gaher, 2005)[3], and the Childhood Trauma Questionnaire (CTQ; 28 items; Bernstein et al., 2003)[4], respectively. The DTS is computed as an average across items (1-5 range), while FFMQ and CTQ is a sum of items (range of 39-195, 25-125). Higher scores indicate greater mindfulness, greater distress tolerance, and greater exposure to childhood trauma. CTQ total scores had 41 missing responses and DTS had 1.

[2] Bohlmeijer, E., ten Klooster, P. M., Fledderus, M., Veehof, M., & Baer, R. (2011). Five facet mindfulness questionnaire--short form. *Mindfulness*.
[3] Simons, J. S., & Gaher, R. M. (2005). The Distress Tolerance Scale: Development and validation of a self-report measure. *Motivation and emotion*, *29*(2), 83-102.
[4] Bernstein, D. P., Stein, J. A., Newcomb, M. D., Walker, E., Pogge, D., Ahluvalia, T., ... & Zule, W. (2003). Development and validation of a brief screening version of the Childhood Trauma Questionnaire. *Child abuse & neglect*, *27*(2), 169-190.

Table S3. Health condition categories.

Gastrointestinal conditions	Neurologic and chronic pain conditions	Inflammatory conditions	Psychological conditions	Other health conditions
• Acid reflux<br>• Barrett's esophagus without dysplasia<br>• Caffeine intolerance<br>• Celiac disease<br>• Cholecystectomy<br>• Colon polyps<br>• Complications from esophagectomy<br>• Dairy intolerance<br>• Diverticulitis<br>• Diverticulosis<br>• Dyspepsia<br>• Eosinophilic esophagitis<br>• Gastroparesis<br>• Gastritis<br>• Gastroesophageal reflux disease<br>• Gilbert syndrome<br>• Gluten sensitivity<br>• Hemochromatosis<br>• Hiatal hernia<br>• Liver disease<br>• Non-alcoholic cirrhosis	• Carpal tunnel syndrome<br>• Chiari malformation<br>• Chronic migraines<br>• Chronic pain<br>• Congenital myasthenic syndrome<br>• Degenerative disc disease with stenosis<br>• Delayed sleep phase circadian rhythm disorder<br>• Dysautonomia<br>• Fibromyalgia<br>• Herniated disc<br>• History of seizures<br>• Idiopathic hypersomnia<br>• Idiopathic intracranial hypertension<br>• Idiopathic length-dependent small-fiber neuropathy<br>• Interstitial cystitis<br>• Joint pain	• Allergies (seasonal, dust, pet)<br>• Ankylosing spondylitis<br>• Asthma<br>• Axial spondyloarthritis<br>• Bursitis of right hip<br>• Dermatomyositis<br>• Endometriosis<br>• Eczema<br>• Exercise-induced asthma<br>• Food allergies<br>• Graves' disease<br>• Hashimoto's thyroiditis<br>• Hidradenitis suppurativa<br>• Lupus<br>• Mast cell activation syndrome<br>• Mixed connective tissue disease<br>• Osteoarthritis<br>• Psoriasis<br>• Psoriatic arthritis	• Agoraphobia<br>• Anxiety<br>• Attention-deficit/hyperactivity disorder<br>• Autism spectrum disorder<br>• Bipolar II disorder<br>• Binge eating disorder<br>• Borderline personality disorder<br>• Complex post-traumatic stress disorder<br>• Dissociative disorder<br>• Insomnia<br>• Major depressive disorder<br>• Obsessive-compulsive disorder<br>• Panic attacks<br>• Post-traumatic stress disorder<br>• Premenstrual dysphoric disorder	• Amputated left toe<br>• Anemia<br>• Benign prostatic hyperplasia<br>• Chronic fatigue<br>• Coronary artery disease<br>• Dry eye disease<br>• Dyslipidemia<br>• Dysmenorrhea<br>• Ehlers-Danlos syndrome<br>• Emphysema<br>• Factor V Leiden clotting disorder<br>• Factor XI deficiency<br>• Familial hypercholesterolemia<br>• Frozen right shoulder<br>• Glucose-6-phosphate dehydrogenase deficiency<br>• Heart failure due to myocardial infarction from spontaneous coronary artery dissection

• Oat intolerance<br>• Rice sensitivity	• Leukoencephalopathy with calcifications and cysts<br>• Menstrual migraines<br>• Migraines<br>• Migraines with aura<br>• Mild obstructive sleep apnea<br>• Muscle injuries<br>• Myofascial pain syndrome<br>• Narcolepsy with cataplexy<br>• Nerve pain due to herniated disc<br>• Neuropathy<br>• Partial idiopathic epilepsy<br>• Pelvic pain<br>• Postural orthostatic tachycardia syndrome<br>• Pulsatile tinnitus<br>• Sleep apnea<br>• Spinal stenosis<br>• Temporomandibular joint dysfunction<br>• Tourette syndrome	• Raynaud's syndrome<br>• Reactive airway disease<br>• Rheumatoid arthritis<br>• Rosacea<br>• Samter's triad (aspirin-exacerbated respiratory disease)<br>• Sinus problems<br>• Sjögren's syndrome<br>• Scleroderma<br>• Specific antibody deficiency<br>• Tendinitis	• Premenstrual syndrome	• High cholesterol<br>• Hypertension<br>• Hypermobility spectrum disorder<br>• Hypogonadism<br>• Hypothyroidism<br>• Infertility<br>• Iron deficiency<br>• Kidney stones<br>• Left hip dysplasia<br>• Metabolic syndrome<br>• Mitochondrial disease<br>• Osteoporosis<br>• Overactive bladder<br>• Prediabetes<br>• Pregnancy<br>• Respiratory infection<br>• Scoliosis<br>• Thyroid cancer<br>• Type 1 diabetes<br>• Type 2 diabetes<br>• Urgency urinary incontinence<br>• Vitreous floaters

Note. The health conditions listed above were derived from participants' open-ended responses collected at baseline. Two participants reported a history of major gastrointestinal surgery (one cholecystectomy and one esophagectomy).

Table S4. Raw descriptive statistics and correlations of continuous predictors.

Variable	Skewness (Kurtosis)	M (SD)	1	2	3	4
1. Age	–	34.22 (13.74)	–			
2. Chronic stress (PSS)	-.01 (2.75)	23.51 (5.34)	-.12*	–		
3. Chronic symptom severity (IBS-SSS)	-.04 (2.71)	274.75 (76.37)	-.03	.11*	–	
4. Momentary stress	.57 (2.65)	3.03 (1.53)	-.07***	.19***	.08***	–
5. Momentary symptom severity	1.31 (4.71)	2.08 (1.21)	-.09***	.03*	.22***	.38***

Note. Correlations among age, chronic stress, and chronic symptom severity were calculated using all available baseline data. Correlations involving momentary stress or momentary symptom severity were calculated using available EMA data.
$^{*}p < .05$. $^{**}p < .01$. $^{***}p < .001$.

Table S5. Distribution of participants by number of completed EMAs.

EMAs Completed	*n* (Stress)	*n* (Symptom Severity)
0	5	5
1	1	1
2	2	2
3	0	0
4	1	1
5	0	0
6	0	0
7	2	2
8	1	1
9	4	4
10	4	3
11	3	4
12	4	4
13	8	7
14	17	17
15	15	16
16	17	15
17	34	34
18	44	40
19	59	64
20	67	66
21	69	71

Note. There was 15.9% of missingness for stress (n = 1,190), 15.7% for symptom severity (n = 1,174).

Figure S2. Individual trajectories of momentary stress and symptom severity.

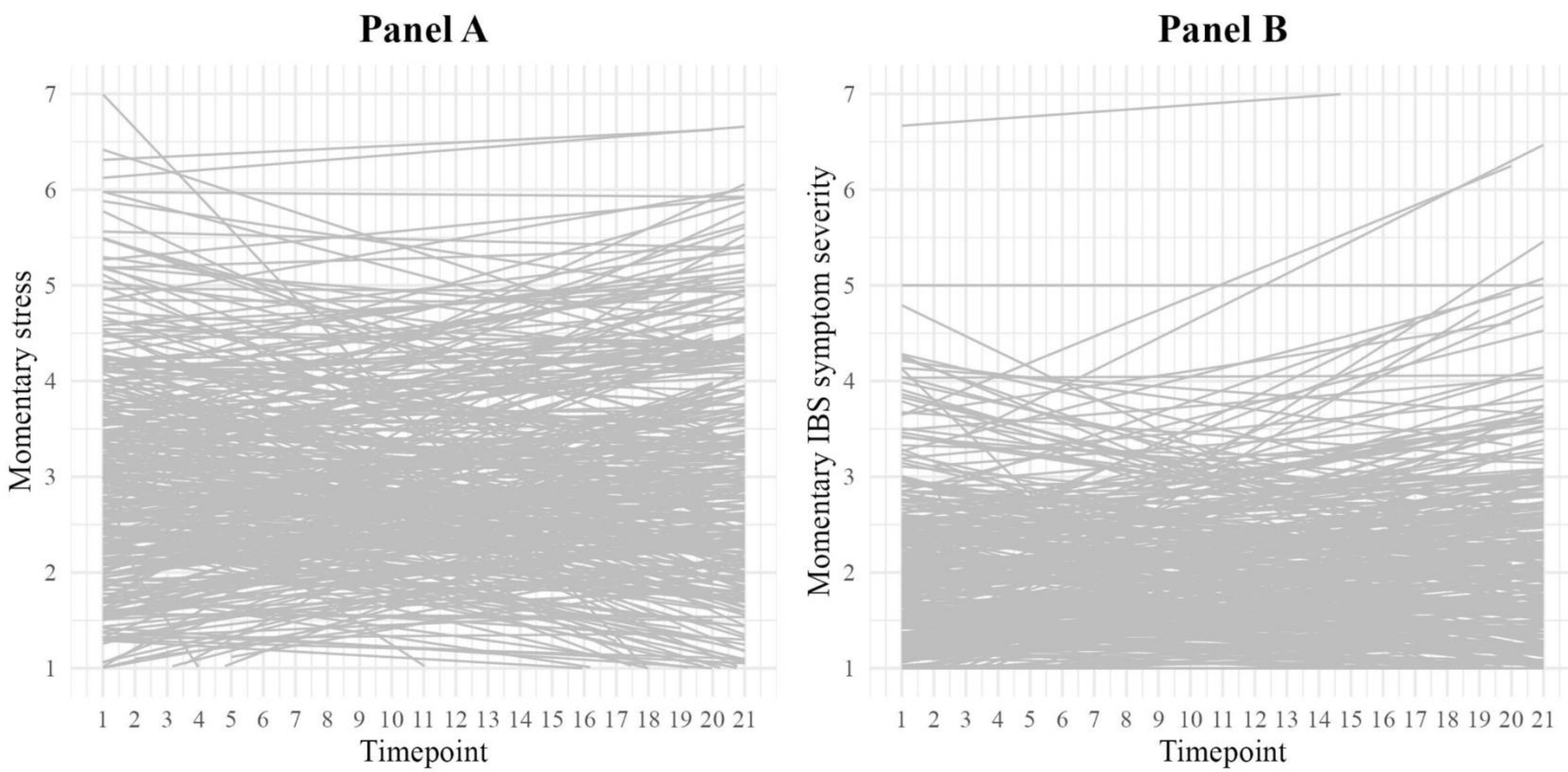

Note. Panel A displays individual participant trajectories of momentary stress scores across EMA timepoints, while Panel B displays momentary symptom severity. These trajectories illustrate the variability in stress and symptom severity patterns over time, ranging from relatively flat profiles to highly fluctuating stress and symptoms. Timepoint refers to the EMA ordinal sequence (ranging from 1 to 21) completed by each participant.

Figure S3. Example individual trajectory of momentary stress and symptom severity.

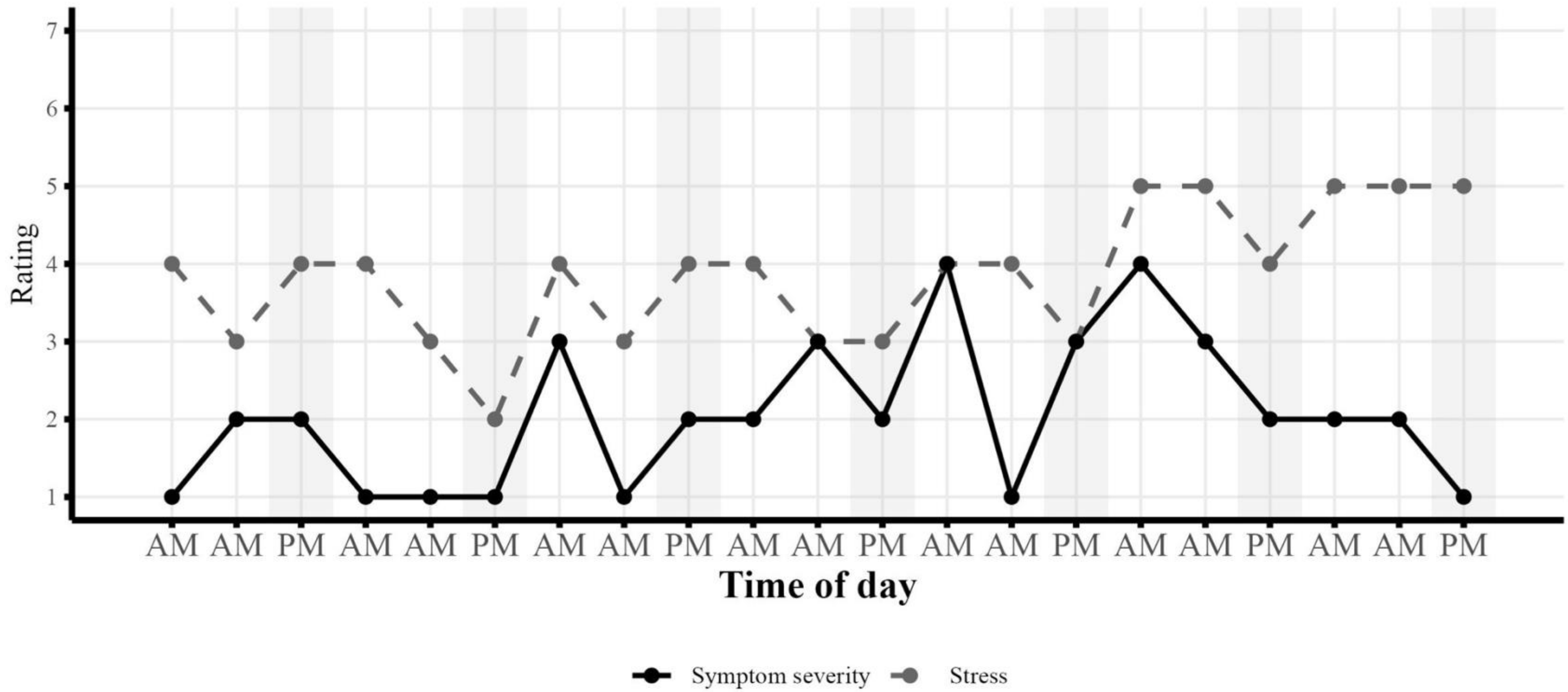

Note. This figure displays within-day and day-to-day variability of momentary stress and symptom severity across the 21 EMA timepoints for an individual participant, with labels indicating time of day (AM = morning and afternoon assessments; PM = evening assessments). Gray shaded regions denote evening EMAs.

EMA Results.

Descriptives. 70.4% of morning surveys occurred between 6:00 a.m. and < 12:00 p.m., 89.8% of afternoon surveys occurred between 12:00 p.m. and < 6:00 p.m., and 74.4% of evening surveys occurred between 6:00 p.m. and < 12:00 a.m. Out-of-window surveys likely reflected technical glitches or participants manually adjusting application settings. Study staff monitored adherence and contacted participants when engagement dropped (e.g., if a full day of EMAs was missed).

Table S6. Estimates of average and momentary stress on symptom severity.

Predictors	**B (SE)**
Afternoon (vs. morning)	**-0.07 (0.02), *p* = .004***
Evening (vs. morning)	-0.03 (0.03), *p* = .31
Day	**0.02 (0.01), *p* = .001***
Weekday (vs. Weekend)	**-0.08 (0.03), *p* = .004***
Age	**-0.01 (0.00), *p* = .01***
Female gender (vs. Male)	-0.03 (0.21), *p* = .90
Nonbinary, genderqueer, or nondisclosed gender (vs. Male)	-0.23 (0.23), *p* = .31
Female sex at birth (vs. Male)	-0.01 (0.20) *p* = .98
Some college (vs. high school)	-0.07 (0.13), *p* = .58
College graduate (vs. high school)	-0.21 (0.12), *p* = .07†
Advanced degree (vs. high school)	**-0.28 (0.12), *p* = .03***
IBS-D (vs. IBS-C)	**-0.47 (0.10), *p* < .001***
IBS-M (vs. IBS-C)	**-0.26 (0.08), *p* = .002***
Chronic stress	–
Chronic symptom severity	–
**Average stress**	**0.36 (0.03), *p* < .001***
**Momentary stress**	**0.28 (0.02), *p* < .001***

Note. *p < .05. † p < .10. The primary model included 6,307 observations (*n* = 352). Average stress: CI [0.29, 0.42]; momentary stress: CI [0.24, 0.31].

Table S7. Estimates of prior stress on subsequent moment severity.

Predictors	**Model 1**<br>**Primary model**<br>**B (SE)**	**Model 2**<br>**+ chronic measures**<br>**B (SE)**	**Model 3**<br>**+ prior severity**<br>**B (SE)**
Afternoon (vs. morning)	**-0.09 (0.03), *p* = .01***	**-0.09 (0.03), *p* = .01***	**-0.09 (0.03), *p* = .01***
Evening (vs. morning)	**-0.09 (0.03), *p* = .01***	**-0.09 (0.03), *p* = .01***	**-0.09 (0.03), *p* = .01***
Day	**0.03 (0.01), *p* < .001***	**0.03 (0.01), *p* < .001***	**0.03 (0.01), *p* < .001***
Weekday (vs. Weekend)	-0.01 (0.03), *p* = .68	-0.01 (0.03), *p* = .66	0.00 (0.03), *p* = .99
Age	**-0.01 (0.00), *p* = .01***	**-0.01 (0.00), *p* = .01***	**-0.01 (0.00), *p* = .01***
Female gender (vs. Male)	0.00 (0.26), *p* = .99	0.03 (0.25), *p* = .90	0.06 (0.25), *p* = .81
Nonbinary, genderqueer, or nondisclosed gender (vs. Male)	-0.28 (0.29), *p* = .33	-0.18 (0.27), *p* = .51	-0.15 (0.27), *p* = .59
Female sex at birth (vs. Male)	-0.08 (0.25), *p* = .76	-0.22 (0.24), *p* = .36	-0.26 (0.24), *p* = .29
Some college (vs. high school)	0.08 (0.16), *p* = .61	0.08 (0.16), *p* = .60	0.04 (0.16), *p* = .79
College graduate (vs. high school)	0.00 (0.15), *p* = .997	0.10 (0.14), *p* = .46	0.07 (0.14), *p* = .62
Advanced degree (vs. high school)	-0.11 (0.15), *p* = .47	0.01 (0.15), *p* = .94	-0.03 (0.15), *p* = .86
IBS-D (vs. IBS-C)	**-0.52 (0.12), *p* < .001***	**-0.44 (0.11), *p* < .001***	**-0.43 (0.11), *p* < .001***
IBS-M (vs. IBS-C)	**-0.27 (0.11), *p* = .01***	**-0.24 (0.10), *p* = .02***	**-0.25 (0.10), *p* = .01***
Chronic stress	–	0.00 (0.01), *p* = .85	0.00 (0.01), *p* = .73
Chronic symptom severity	–	**0.00 (0.00), *p* < .001***	**0.00 (0.00), *p* < .001***
Prior severity	–	–	**0.13 (0.02), *p* < .001***
**Prior stress**	**0.06 (0.01), *p* < .001***	**0.06 (0.01), *p* < .001***	0.02 (0.01), *p* = .07†

Note. *p < .05. † p < .10. All models included 5,340 observations (*n* = 350). Prior stress: Model 1 and 2 CI [0.04, 0.09]; Model 3 CI [–0.00, 0.05].

Table S8. Estimates of prior symptom severity on subsequent stress.

Predictors	**Model 1 Primary model B (SE)**	**Model 2 + chronic measures B (SE)**	**Model 3 + prior stress B (SE)**
Afternoon (vs. morning)	0.05 (0.04), *p* = .20	0.05 (0.04), *p* = .20	0.03 (0.04), *p* = .51
Evening (vs. morning)	**-0.14 (0.04), *p* = .001***	**-0.14 (0.04), *p* = .001***	**-0.18 (0.04), *p* < .001***
Day	**0.02 (0.01), *p* = .01***	**0.02 (0.01), *p* = .01***	**0.03 (0.01), *p* = .002***
Weekday (vs. Weekend)	**0.33 (0.04), *p* < .001***	**0.33 (0.04), *p* < .001***	**0.24 (0.04), *p* < .001***
Age	**-0.01 (0.00), *p* = .03***	-0.01 (0.00), *p* = .08†	-0.01 (0.00), *p* = .14
Female gender (vs. Male)	0.21 (0.37), *p* = .57	0.22 (0.35), *p* = .53	0.27 (0.35), *p* = .44
Nonbinary, genderqueer, or nondisclosed gender (vs. Male)	0.25 (0.40), *p* = .54	0.30 (0.39), *p* = .44	0.37 (0.38), *p* = .33
Female sex at birth (vs. Male)	-0.19 (0.36), *p* = .60	-0.25 (0.35), *p* = .47	-0.34 (0.34), *p* = .32
Some college (vs. high school)	0.13 (0.23), *p* = .57	0.06 (0.22), *p* = .78	0.11 (0.22), *p* = .61
College graduate (vs. high school)	0.19 (0.20), *p* = .34	0.21 (0.20), *p* = .30	0.28 (0.19), *p* = .15
Advanced degree (vs. high school)	0.20 (0.22), *p* = .36	0.25 (0.21), *p* = .23	0.32 (0.21), *p* = .12
IBS-D (vs. IBS-C)	0.03 (0.17), *p* = .84	0.06 (0.16), *p* = .72	0.06 (0.16), *p* = .70
IBS-M (vs. IBS-C)	-0.03 (0.15), *p* = .82	-0.05 (0.14), *p* = .74	-0.05 (0.14), *p* = .71
Chronic stress	–	**0.05 (0.01), *p* < .001***	**0.05 (0.01), *p* < .001***
Chronic symptom severity	–	**0.00 (0.00), *p* = .04***	**0.00 (0.00), *p* = .03***
Prior stress	–	–	**0.17 (0.02), *p* < .001***
**Prior severity**	**0.08 (0.02), *p* < .001***	**0.08 (0.02), *p* < .001***	0.01 (0.02), *p* = .66

Note. *p < .05. † p < .10. Models 1–2 included 5,340 observations (*n* = 350); Model 3 included 5,327 observations (*n* = 350). Prior severity: Model 1 and 2 CI [0.04, 0.11]; Model 3 CI [–0.03, 0.05].
